# Development and Evaluation of a Monoclonal Antibody-Based Blocking Enzyme-Linked Immunosorbent Assay for the Detection of Antibodies against Novel Duck Reovirus in Waterfowl Species

**DOI:** 10.1128/spectrum.02581-22

**Published:** 2022-11-29

**Authors:** Tao Yun, Jionggang Hua, Weicheng Ye, Liu Chen, Zheng Ni, Yinchu Zhu, Cun Zhang

**Affiliations:** a State Key Laboratory for Managing Biotic and Chemical Threats to the Quality and Safety of Agro-products, Institute of Animal Husbandry and Veterinary Sciences, Zhejiang Academy of Agricultural Sciences, Hangzhou, China; Changchun Veterinary Research Institute

**Keywords:** duck novel reovirus (NDRV), monoclonal antibody, blocking ELISA, antibody detection, waterfowl species

## Abstract

The novel duck reovirus (NDRV) is an emerging pathogen that causes disease in various waterfowl species. Since the outbreak, it has caused huge economic losses to the duck industry in China. A rapid, reliable, and high-throughput method is required for epidemiological investigation and evaluation of vaccine immunogenicity. A good first step would be establishing an enzyme-linked immunosorbent assay (ELISA) that could detect NDRV antibodies in different breeds of ducks and geese from the serum and egg yolk. This study used a recombinant NDRV σB protein and a corresponding horseradish peroxidase (HRP)-labeled monoclonal antibody to develop a blocking ELISA (B-ELISA). The cutoff value of the B-ELISA was 37.01%. A total of 212 serum samples were tested by the B-ELISA, and the virus neutralization test (VNT) was the gold standard test. The sensitivity and specificity of the B-ELISA were 92.17% (106/115) and 97.94% (95/97), respectively. The agreement rates between the B-ELISA and VNT were 94.81% (kappa value, 0.896). The B-ELISA could specifically recognize anti-NDRV sera without cross-reacting with other positive serums for other major diseases in ducks and geese. The inter- and intra-assay coefficients of variation (CVs) of the B-ELISA and VNT assays were acceptable. In conclusion, the novel B-ELISA could be a rapid, simple, safe, and economically attractive alternative to the VNT in assessing duck flocks’ immunity status and in epidemiological surveillance in multiple waterfowl species.

**IMPORTANCE** NDRV disease is a new epidemic disease in waterfowl that first appeared in China. Compared with the classical DRV (CDRV), NDRV is associated with more severe symptoms, a higher mortality rate, and a broader host range. NDRV has become the prevalent genotype in China. At present, there are no commercially available diagnostic products for the NDRV disease. VNT, as the gold standard serologic test, is not only time-consuming and laborious, but also has high requirements for facilities and equipment, which is not suitable for clinical application. Conventional ELISA requires specific antispecies conjugates that are not currently available. B-ELISA not only has the advantage of higher analysis specificity, but also can be used to test specific antibodies against different waterfowl species, because no species-specific conjugates are required in such detection. Therefore, it is necessary to establish a B-ELISA for the detection of antibodies against NDRV in waterfowl species.

## INTRODUCTION

Duck reovirus (DRV) is an important waterfowl pathogen that belongs to the avian *Orthoreovirus* species group II in the genus *Orthoreovirus*, family *Reoviridae* (1, 2). Based on the necropsy lesions and phylogenetic analysis, DRV can be classified as genotype I (classical duck reovirus, CDRV) and genotype II (novel duck reovirus, NDRV) (3, 4). CDRV (also known as the Muscovy duck reovirus) mainly infects the Muscovy duck and goose and was first isolated in France (5, 6). The disease is characterized by small white necrotic foci covering the surface of the liver and spleen (5–7). The disease spread to China in 1997 and became widely prevalent in southern China’s major Muscovy duck production area (8). NDRV can infect almost all kinds of waterfowl species (domestic ducks and geese) (9). The main lesions are severe necrosis and hemorrhagic foci of the liver and spleen (known locally as hemorrhagic-necrotic hepatitis). The disease first occurred in 2000, spread quickly from south to north in eastern China, and eventually became an epidemic disease endangering the health of waterfowl worldwide (9–14).

NDRV is a segmented double-stranded RNA virus comprising 10 double-stranded RNA fragments. Based on the electrophoretic mobility of RNAs of different sizes, the fragments can be classified into three groups: large (L), medium (M), and small (S). There are three large fragments, L1, L2, and L3, encoding three proteins, i.e., λA, λB, and λC, respectively. There are three medium fragments, M1, M2, and M3, encoding μA, μB, and μNS, respectively. Finally, there are four small fragments, S1, S2, S3, and S4, encoding six proteins, i.e., σA, σB, σC, σNS, P10, and P18 (4, 10, 14). The S3-encoded protein σB is the main component of the outer capsid protein of NDRV, with a function similar to that of the σ3 protein of the mammalian reovirus (MRV) and the σB protein of the avian reovirus (ARV) and CDRV. NDRV and CDRV are different genotypes of DRV, which are members of the avian *Orthoreovirus* species with ARV. The amino acid homology of the NDRV σB protein with ARV and CDRV is about 60% and 70%, respectively. The NDRV σB protein contains group-specific neutralizing epitopes and generates a strong antibody response against the virus. Furthermore, the sequence of NDRV σB is relatively conserved and closely related to virus infection, pathogenicity, and immunogenicity. Therefore, it is a suitable candidate for serodiagnosis of the NDRV (15, 16).

Currently, the routine detection methods for the NDRV pathogen and associated antibodies include virus isolation (4, 10, 14), RT-PCR (17, 18), virus neutralization test (VNT) (13), and indirect enzyme-linked immunosorbent assay (ELISA) (19). ELISA is a powerful technique for detecting and measuring antigens and antibodies (20). This method has advantages such as ease of operation and high sensitivity and specificity, making it much more suitable for assessing immune responses and epidemiological surveys.

The NDRV disease has spread rapidly since the outbreak and has had large-scale occurrences in the main duck-producing areas of China. It has been reported that the host range of NDRV has been enlarged. All species of ducklings (Muscovy duck, semi-Muscovy duck, Cherry Valley duck, Peking duck, Ma duck, and wild duck) and goslings can be infected (3, 4, 10, 11, 21, 22), and genomic variation has already occurred (9, 23–26). The disease has seriously hindered the healthy development of the waterfowl industry in China. The detection, surveillance, prevention, and control measures for the NDRV disease are of great significance for the duck industry. There is currently no commercially available universal anti-IgY-conjugated secondary antibody against the waterfowl species. Therefore, conventional ELISA can not simultaneously detect NDRV antibodies of different waterfowl species.

Therefore, this paper describes a blocking ELISA (B-ELISA) for the serological diagnosis of sera from any waterfowl species, using a purified recombinant NDRV σB protein as the coating antigen and horseradish peroxidase (HRP)-labeled anti-σB protein monoclonal antibody as the detection antibody. This method proved to be a rapid, simple, safe, and lower-cost approach for the high throughput of clinical serum samples.

## RESULTS

### Expression of the NDRV σB recombinant protein.

The recombinant plasmid pET-SUMO-SigB with a His tag was transformed into Escherichia coli BL21(DE3), and the recombinant σB protein was identified as being approximately 55 kDa by SDS-PAGE analysis (see Fig. S1A in the supplemental material), consistent with the expected size of the His-SUMO-σB recombinant protein. After purification with an Ni-NTA kit (Qiagen, Valencia, CA, USA), the purified protein was dialyzed and renatured in renaturation buffer, and the purified His-SUMO-σB protein was then detected with duck anti-NDRV polyclonal serum. Western blot analysis showed that the purified His-SUMO-σB proteins reacted specifically with duck anti-NDRV polyclonal antibody with an approximate molecular mass of 55 kDa (Fig. S1B). This indicates that recombinant His-SUMO-σB protein was successfully expressed and could be used as an immunogen for monoclonal antibody production using BALB/c mice.

### Preparation and identification of monoclonal antibody (MAb) against NDRV σB.

**FIG 1:**
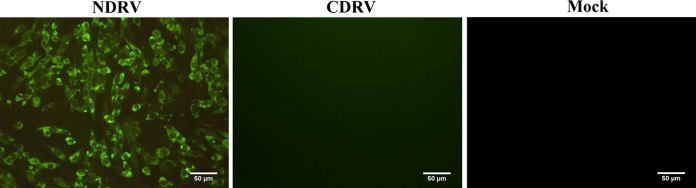
Immunofluorescence analysis of MAb 2-C10 with NDRV- and CDRV-infected DF-1 cells. DF-1 cells inoculated with NDRV ZJ00M and CDRV ZJ2000M (MOI, 0.01) and stained with 2-C10- and FITC-conjugated goat anti-mouse IgG at 48 h postinoculation (left and middle, respectively) and (right) uninfected DF-1 cells are shown (scale bar, 50 μm).

The purified recombinant SUMO-σB protein was used to immunize 6-week-old BALB/c mice to prepare MAbs recognizing the σB protein of NDRV. After cell fusion, subcloning, and screening of hybridoma cells (27), a hybridoma stably secreting MAb against NDRV was obtained and designated as 2-C10. The results of antibody isotyping showed that 2-C10 belonged to IgG1, with a kappa (κ) light chain. Using a Western blot, 2-C10 showed reactivity with the σB protein on the virion of NDRV ZJ00M (Fig. S1C). Strong green fluorescence in an indirect immunofluorescence assay (IFA) was observed in the cytoplasm of chicken embryo fibroblasts (DF-1) infected with NDRV ZJ00M (Fig. 1). No green fluorescence was observed in DF-1 infected with CDRV ZJ2000M or uninfected DF-1 cells by IFA (Fig. 1). The titer of MAb obtained from purified mouse ascites was determined to be approximately 1:2.4 × 10^6^ by indirect ELISA with rSUMO-SigB as the coated antigen. Subsequently, neutralization capacities of the MAb were characterized, 2-C10 showed neutralization activity, and neutralizing antibody titers (NAT) reached 1:200.

### Establishment of B-ELISA based on NDRV σB protein and 2-C10 MAb.

The optimal concentrations of coated protein (NDRV σB) and HRP-conjugated MAb (HRP-2-C10) (Fig. S2A) revealed that the optimal concentration of NDRV σB protein was 0.3125 μg/mL. The amount of HRP-2-C10 was 0.625 μg/mL. The optical density at 450 nm (OD_450_) value of the B-ELISA was always around 1.2, and the concentration point that achieved the optimal inhibition was within the titration curve (linear range). Different dilutions of 8 serum samples (5 positive and 3 negative samples) (sera VI) were determined by a concentration of HRP-2-C10 in the B-ELISA to optimize serum sample dilution and minimize the required serum volume and background noise. Finally, a 1:5 serum dilution was chosen as optimal (Fig. S2B). In summary, these conditions were used in all subsequent B-ELISA experiments. The cutoff level for the B-ELISA was tested using B-ELISA, which showed that the mean percent inhibition (PI) value for these sera (sera I) was −0.64%, with a standard deviation (SD) of 12.55%. Therefore, the cutoff value for the developed B-ELISA was set at a PI value of 37.01% based on the criteria, which was the mean PI value plus 3 × SD.

### Evaluation of the B-ELISA.

To evaluate the specificity of the newly developed B-ELISA, 40 sera from classical DRV (CDRV)-infected duck (*n* = 5), duck Tembusu virus (DTMUV)-infected duck (*n* = 8), duck plague virus (DPV)-infected duck (*n* = 4), goose parovirus (GPV)-infected goose (*n* = 6), Muscovy duck parovirus (MDPV)-infected duck (*n* = 3), avian influenza virus (AIV) subtype H9-infected duck (*n* = 3), duck hepatitis A virus (DHAV-1)-infected duck (*n* = 4), Riemerella anatipestifer-infected duck (*n* = 3) and Pasteurella anatipestifer (PA)-infected duck (*n* = 4) (sera III) were detected. The results showed that the PI values of all sera were lower than the cutoff value, and PI values were negative (less than 20.72%), ranging from −16.43% to 17.04% (Fig. S3). The established B-ELISA could clearly discriminate the NDRV-positive serum from other nonspecific positive duck serum, demonstrating that the B-ELISA is NDRV specific. The repeatability of the B-ELISA was evaluated by calculating the coefficient of variation (CV) of the inter- and intra-assay variation of PI values for 8 serum samples (5 positives and 3 negatives) (sera VI). The interassay CVs were 1.37% to 7.02%, and the intra-assay CVs were 1.62% to 8.69% (Table S1). As both CV values were less than 10%, the newly developed B-ELSIA showed excellent reproducibility.

### Correlation between B-ELISA and VNT.

To assess the diagnostic performance of the developed B-ELISA in relation to the VNT, 212 serum samples (sera II) were tested in parallel by the two tests. The statistical analysis showed that the relative sensitivity and specificity of the B-ELISA were 92.17% and 97.94%, respectively. The rate of coincidence of the B-ELISA and VNT was 94.81% (201/212), which showed high agreement between the B-ELISA and VNT (kappa value, 0.896; Table 1). The results confirmed that this B-ELISA was highly accurate.

**TABLE 1:** Comparison of the results of the blocking ELISA and the VNT^*a*^ based on 212 field sera

Blocking ELISA	Virus neutralization test (VNT)		
	Positive serum (*n*)	Negative serum (*n*)	Total
Positive serum	106	2	108
Negative serum	9	95	102
Total	115	97	212

a Relative specificity, 106 of 115, or 92.17%. Relative sensitivity, 95 of 97, or 97.94%.

### Application of B-ELISA in field samples.

**(i) Evaluation of vaccine immunity.** The B-ELISA was used to detect 1,038 Muscovy duck serum and 540 Muscovy duck egg yolk samples (sera IV) within 1 to 6 months after the booster vaccination with NDRV inactivated vaccine. The results showed that the positive rate of serum antibodies was 94.5%, and the positive rate of yolk antibodies was 91.9% (Table 2). In addition, the anti-NDRV antibodies in the egg yolk were not detected until the second week after the first immunization. The positive rate was 30% to 40%, which continued to increase. In contrast, the positive rate and the PI value peaked at week 3 after the booster immunization (data not shown). These results showed that the NDRV inactivated vaccine induced the production of antibodies in laying Muscovy ducks but also ingested maternal antibodies into the yolk (28, 29).

**TABLE 2:** Detection of NDRV antibody in field serum and egg yolk samples from vaccinated breeding Muscovy ducks by blocking ELISA

Blocking ELISA	Positive (*n*)	Negative (*n*)	Positive rate (%)
Breeding Muscovy duck	979	59	94.5
Egg yolk	496	44	91.9

### Detection of antibodies in field serum samples.

The B-ELISA method was performed on 606 Muscovy duck serum samples, 60 Cherry Valley duck serum samples, 100 laying duck serum samples, and 96 goose serum samples from different farms (sera V) and from birds that were not vaccinated with autogenous NDRV inactivated vaccine. As shown in Table 3, the positive rate of NDRV antibodies in commercial Muscovy ducks was 8.5% (9/106), and it was 22.6% (113/500) in breeding Muscovy ducks. The total detection rate of positive antibodies in Muscovy ducks was 18.6% (122/606).

**TABLE 3:** Detection of NDRV antibody in field serum samples of Muscovy duck, Cherry Valley Duck, laying duck and goose by B-ELISA

Waterfowl species	Positive (*n*)	Negative (*n*)	Positive rate (%)
Muscovy duck			
Commercial Muscovy duck	9	97	8.5
Breeding Muscovy duck	113	387	22.6
Cherry Valley duck	4	56	6.7
Laying duck	6	94	6.0
Goose	8	88	8.3

## DISCUSSION

NDRV disease is a new epidemic disease caused by a new genotype of DRV. The disease has spread rapidly since the outbreak and has had large-scale occurrences in the main duck-producing areas of China. At present, there are no commercially available diagnostic products for NDRV disease. Therefore, it was essential to develop a diagnostic assay for the detection of antibodies specific to NDRV. This study used B-ELISA with the purified recombinant NDRV σB protein as the coating antigen and HRP-labeled anti-σB protein monoclonal antibody as the detection antibody. The results indicated that the developed B-ELISA would be rapid, simple, safe, and lowcost-effective compared to VNT in assessing duck flocks’ immunity status and conducting epidemiological surveillance in multiple waterfowl species.

The major outer capsid protein of NDRV is σB, which has antigenicity and can induce host-protective antibodies (15, 16, 30, 31) and can also induce group-specific neutralizing antibody (16, 30, 32). It was previously reported that the most conserved amino acid in the deduced amino acid sequence of σB for avian orthoreovirus is in the N-terminal region (1 to 113 amino acids [aa]) (30, 31). We developed a hybridoma that stably secreted MAb against NDRV and designated it 2-C10. We then confirmed that the epitope of MAb 2-C10 was also located in the N-terminal region of σB. However, it was found that the epitope was not conserved between NDRV and CDRV by alignment of the amino acid sequences (results not shown). Furthermore, MAb 2-C10 could specifically bind to native NDRV and recombinant σB antigens. However, it does not react to CDRV in IFA (Fig. 1) and Western blotting and has only neutralizing activity against NDRV. These results indicated that the MAb 2-C10 could specifically block NDRV-induced neutralizing antibodies in waterfowl and could be used as a blocking antibody in B-ELISA.

This study developed a B-ELISA to detect NDRV serum antibodies from waterfowl species based on the anti-NDRV σB MAb. The σB as a coating antigen was expressed in prokaryotic systems. It was simple to operate and easy to prepare in large quantities. The MAb with blocking effect was screened by monoclonal antibody techniques, which could react with prokaryotic expressed σB and native NDRV simultaneously. For a shorter turnaround time and lower cost, the MAb 2-C10 was conjugated with HRP as the detection antibody, which eliminated the step of using an anti-mouse immunoglobulin conjugate (33, 34). It could reduce the protocol time by at least 1 h. The HRP-conjugated MAb could compete with the NDRV-specific antibody in the serum to bind to σB antigen protein to minimize the occurrence of nonspecific reactions and improve the specificity of detection. As a detection antibody, the MAb conjugated with HRP could overcome the limitation of traditional ELISA, which required specific anti-waterfowl species conjugates (35).

Currently, the main serological detection assays of NDRV-specific antibodies are the VNT and the conventional ELISA method (19, 36, 37). VNT is not only the most specific method for serological diagnosis, but also the gold standard for the evaluation of antibody titer. However, there are some limitations, including that it uses live virus stocks, it is time-consuming and laborious, it has poor repeatability, and it is not suitable for assaying a large number of samples at one time, which limits the routine applicability of the VNT (37–42). Moreover, although the conventional ELISA is simple and rapid (20), it is unsuitable for large-scale screening of NDRV disease in waterfowl species, mainly due to the fact that the routine ELISA requires species-specific conjugates for the detection of NDRV antibodies in waterfowl species, which are currently unavailable (35, 43).

It was reported that sample quality had an adverse effect on serologic test results, such as microbial contamination, hemolysis, hyperlipidemia, etc. (35, 44). As shown in Table 1, nine samples with inconsistent results of VNT and B-ELISA were of inferior quality. Of these nine sera, six scored as negative by B-ELISA and as positive by VNT, and the PI values of these six sera were close to the cutoff value (data not shown). The results of the remaining 203 serum samples were completely consistent. Comparing the B-ELISA results with VNT showed that the agreement rate of the two methods was 94.81% (201/212) and showed a high agreement (κ = 0.896). This indicated a perfect concordance between the two methods, and this novel B-ELISA could replace the VNT.

The ideal diagnostic method should have high sensitivity, specificity, and good repeatability (45). The B-ELISA was found to be specific to NDRV. There were no cross-reactions with the sera that were strongly positive for other waterfowl viruses and bacteria, especially for the positive sere of CDRV belonging to DRV, demonstrating that the specificity of the B-ELISA was extremely high. In addition, it was reported that the coefficient of variation of the original absorbance value is less than 20%, indicating sufficient repeatability (46). The repeatability results of this study showed that the intra-assay CVs and the interassay CVs of the B-ELISA were lower than 10%, so this B-ELISA also had excellent repeatability. In addition, as shown in Table 3, the positive rates of NDRV antibodies in Cherry Valley ducks, laying ducks, and geese were 6.7% (4/60), 6.0% (6/100), and 8.3% (8/88), respectively. This revealed that the positive rate of NDRV antibody in the sera of Muscovy ducks, Cherry Valley ducks, and geese was different on nonvaccinated farms, and the natural infection rate of the breeding Muscovy duck was the highest. Therefore, breeder waterfowl should be vaccinated during the prelaying to early-laying period to protect their offspring from NDRV infection.

In conclusion, we established a B-ELISA based on σB MAb for detecting NDRV antibodies from any waterfowl species. It has a high diagnostic sensitivity and specificity. This B-ELISA could be a very valuable tool for serological surveillance of NDRV infection in multiple waterfowl species for and monitoring the efficiency of vaccination.

## MATERIALS AND METHODS

### Ethics statement.

All animal experiments were carried out in accordance with the Regulations of the Administration of Affairs Concerning Experimental Animals approved by the State Council of China. Animal care and protocols were approved by the Research Ethics Committee of ZAAS (permit number ZAAS2020016, March 2020). The animals were humanely bled and then euthanized by intravenous injection of sodium pentobarbital at the end of the experiment.

### Study samples.

**(i) Naive samples.** A total of 521 samples obtained from the healthy waterfowl from different farms in Zhejiang, China, which had no history of exposure to NDRV or vaccination, were used to establish the cutoff value. The serum from this criterion that showed the lowest blocking rate was chosen as the negative control for the subsequent tests.

**(ii) Serum samples from infected ducks.** A total of 212 serum samples collected from breeding duck farms that were confirmed to be infected with NDRV by real-time quantitative reverse transcriptase PCR (RT-PCR) in the previous year (47). The serum with the highest neutralizing antibody titers detected by VNT was used as the positive control for subsequent tests.

**(iii) Serum samples from other pathogen-infected ducks.** In order to evaluate the specificity of the assay, nine positive sera for other known common duck pathogens, including CDRV (*n* = 5), DTMUV (*n* = 8), DPV (*n* = 4), GPV (*n* = 6), MDPV (*n* = 3), AIV subtypes H9 (*n* = 3), DHAV-1 (*n* = 4), R. anatipestifer (*n* = 3), and PA (*n* = 4) were included in this study, which were validated serum samples kept in our laboratory (19).

**(iv) Serum and egg yolk samples from vaccinated ducks.** A total of 1,578 serum and egg yolk samples from vaccinated Muscovy ducks were used in this study. A total of 1,038 serum samples were obtained from ducks at a Muscovy duck breeding farm in Zhejiang that were immunized twice intramuscularly with a lab-made inactivated NDRV ZJ00M plus Marcol 52 mineral oil (ESSO, Paris, France) in a final volume of 2 mL at an interval of 3 weeks. They were collected within 1 week to 4 months after the second immunization. In addition, 540 egg yolk samples were collected within 1 to 6 months after the booster vaccination.

**(v) Field samples.** A total of 862 adult waterfowl samples from Muscovy ducks (*n* = 606), Cherry Valley ducks (*n* = 60), laying ducks (*n* = 100), and geese (*n* = 96) were collected from domestic farms that were not infected with NDRV or immunized with NDRV-inactivated vaccine.

**(vi) Serum samples.** In order to examine the limit of detection of the development B-ELISA, five positive and three negative serum samples were employed, which were selected from the above-described naive samples and serum samples from vaccinated ducks, respectively.

All serum samples were stored frozen at −80°C and inactivated by heating at 60°C in a water bath for 2 h before use in assays as previously described (48).

### Constructing recombinant plasmid.

The full-length σB coding sequence was amplified using the forward primer 5′-CGC*GGATCC*ATGGAGGTGCGTGTGCCA AAC-3′ (Tsingke, China) and the reverse primer 5′-GCA*CTCGAG*TTACCACCT ACACTCCAGGAAG-3′ (Tsingke) based on the S3 segment (GenBank accession number KF154118) of NDRV ZJ00M. The underlined and italicized bases are the sequences for the restriction sites of BamHI and XhoI (TaKaRa Biotechnology [Dalian] Co., Ltd, Dalian, China). The PCR amplicons were cloned into a downstream side of the SUMO gene (pET-28a-SUMO) at the BamHI and XhoI restriction sites and resulted in the recombinant plasmid pET-SUMO-σB. The positive clones were confirmed by restriction digestion followed by DNA sequencing (Biosune Biotechnology Co., Ltd, Shanghai, China).

### Preparation of recombinant NDRV σB protein.

The recombinant plasmid pET-SUMO-σB was transformed into Escherichia coli BL21(DE3) (TransGen Biotech Co., Ltd, Beijing, China) and induced with 1 mM isopropyl-β-d-galactopyranoside (IPTG) at 37°C for 5 h in culture medium. The cell pellets were harvested by centrifugation at 4,000 × *g* for 20 min, resuspended in phosphate-buffered saline (PBS) (0.1 M, pH 7.5), and lysed by ultrasonication (20 kHz) on ice for 15 min. After centrifugation at 12,000 rpm for 30 min at 4°C, the inclusion body (IB) pellet was washed with PBS (0.1 M, pH 8.0) containing 50 mM Tris-HCl, 1 mM EDTA, and 2% Triton X-100. The washed IB pellet was solubilized with binding buffer (20 mM Tris-HCL [pH 8.0], 8 M urea, 500 mM NaCl, and 1 mM β-mercaptoethanol) and stirred for 1 h at room temperature. The suspension was centrifuged at 12,000 rpm for 30 min at 4°C, and the supernatant was collected for further purification. The expressed recombinant protein was purified using Ni-NTA affinity chromatography columns (Qiagen, Valencia, CA, USA) according to the manufacturer’s instructions. The protein concentration was quantified using the Quawell protein assay (Quawell Technology, CA, USA). Finally, SDS-PAGE and Western blotting were used to confirm the expression, purification, and antigenicity of the recombinant NDRV His-SUMO-σB protein (rσB) with the positive duck anti-NDRV polyclonal serum, as previously described (4, 19).

### Generation of MAb to NDRV σB protein.

Using the prime-boost approach (49), three 5- to 6-week-old female BALB/c mice (purchased from Hangzhou Medical College Laboratory Animal Research Center) were immunized subcutaneously with the purified rσB protein (50 μg per mouse) emulsified with complete Freund’s adjuvant (Sigma-Aldrich, St. Louis, MO, USA). Three booster immunizations of the same dose were given at 2-week intervals mixed with an incomplete adjuvant. Three days after the final booster immunization, the spleen cells isolated from immunized mice were fused with myeloma SP2/0 cells by using 50% (wt/vol) polyethylene glycol 1500 (PEG 1500; Boehringer Mannheim, Indiana, USA) at a ratio of 10:1. The hybridoma cells were grown in selective medium (hypoxanthine-aminopterin-thymidine [HAT]; Gibco, New York, USA) for 10 days, and then the hybridoma cells were screened by indirect ELISA (19) and coated with purified rσB protein. The positive hybridoma clones were further subcloned by limiting dilution to establish single stable clones. Briefly, a hybridoma cell suspension was diluted in growth medium (RPMI 1640; Gibco) supplemented with 10% fetal calf serum (FCS) and seeded in 96-well microplates at approximately one cell per well. After 10 days of culture, indirect ELISA was performed to select clones that secreted the desired antibodies. The positive clones were transferred to the culture flasks and propagated with the growth medium. Hybridoma cells were then screened by indirect ELISA, indirect immunofluorescence assay (IFA), and VNT. The NDRV σB-specific MAb, designated 2-C10, was selected by its capacity to compete with antibodies in anti-NDRV-positive control serum in the B-ELISA. Following the manufacturer’s instructions, its subtype was classified using the mouse MAb isotyping kit (Thermo Scientific, USA). The hybridoma was injected into the peritoneal cavities of BALB/c mice. Subsequent ascite fluid was collected and purified by affinity chromatography using the HiTrap protein G column (GE Healthcare Life Sciences, Pennsylvania, USA) (50).

### Western blot analysis.

The binding ability of the MAb 2-C10 to the whole virus was examined by Western blot analysis. The crude viral particles were obtained using a previously described method (4) with a minor modification. The NDRV ZJ00M strain was inoculated by multiple infections (multiplicity of infection [MOI], 0.01) in DF-1 cells. When virus-specific cytopathic effects (CPE) were observed in ≥80% of cells, they were scraped into the medium. They underwent three freeze-thaw cycles, and cellular debris was removed by centrifugation at 10,000 × *g* for 30 min. The virus was concentrated from the supernatant by ultracentrifugation at 130,000 × *g* for 1 h. The crude viral particles were mixed with loading buffer (pH 7.4) (1% SDS, 25 mM Tris-HCl, 0.5% β-mercaptoethanol, and 0.001% bromophenol blue) and then incubated at 105°C for 10 min before being loaded onto the gel. The NDRV lysates were separated on 4% to 20% gradient gels by SDS-PAGE and transferred onto nitrocellulose membrane (Bio-Rad). The membrane was then blocked with a blocking buffer (5% skimmed milk powder in PBS with 0.05% Tween 20) overnight at 4°C. After washing, the MAb 2-C10 was diluted (1:5,000) in the blocking buffer, and the mixture was then incubated for 1 h at 37°C. The membrane was washed three times with PBS with Tween 20 (PBST) and probed with a 1:4,000 dilution of HRP-conjugated goat anti-mouse IgG (Sigma, St. Louis, MO, USA) for 30 min at room temperature. Finally, the membrane was rewashed and developed with chemiluminescence (ECL) reagents (Thermo Fisher Scientific, Rockford, IL, USA). The image was visualized using the Bio-Rad ChemiDocXRS+ chemiluminescence imaging system (Bio-Rad, Hercules, CA, USA).

### IFA.

The specificity of the MAb 2-C10 with NDRV was performed by IFA. The assay was performed using a modification of previously established procedures (38). Briefly, DF-1 cells were cultured in 24-well plates and were infected with NDRV ZJ00M at an MOI of 0.01. At 48 h postinfection, the infected DF-1 cells were washed three times with PBS and then fixed with an ice-cold acetone/methanol (1:1) mixture for 30 min at −20°C. The fixed cells were incubated with the MAb 2-C10 (diluted 1:2,000) at 37°C for 1 h. After washing three times, fluorescein isothiocyanate (FITC)-conjugated goat anti-mouse IgG (Sigma, St. Louis, MO, USA) was added at a 1:500 dilution and incubated at 37°C for 1 h in the dark. Finally, the cells were washed three times in PBST and air-dried for 10 min at room temperature and finally observed under a fluorescent microscope.

### VNT.

The VNT was performed in 96-well tissue culture microplates, as previously described, to determine the antibody specificities for diagnostic serum samples and quantitated antibody titers (30). In brief, the heat-inactivated sera were first diluted 5-fold and then serially diluted 2-fold (in duplicate) in 96-well plates with cell culture medium. Then, 100 50% tissue culture infective doses (TCID_50_s) of NDRV ZJ00M was mixed with an equal volume of diluted sera. The virus-serum mixtures were incubated at 37°C for 1 h in 5% CO_2_. Subsequently, 5 × 10^4^ DF-1 cells were added to each of the wells as indicators of residual infectivity. A series of control wells (in duplicate) for 0.1, 1, 10, and 100 TCID_50_ virus and virus-free cells were set up on each plate. The plates were incubated at 37°C in 5% CO_2_ for 96 h. The plate was checked by observing the cell monolayer under an inverted microscope for virus-specific CPE. Neutralizing antibody titers (NAT) were expressed as the reciprocal of the final dilution of sera that caused a 50% reduction in CPE in duplicate wells.

### HRP conjugation of MAb 2-C10.

The purified MAb 2-C10 was conjugated to HRP with the HRP Lightning-Link kit (Abcam, Cambridge, UK) according to the manufacturer’s instructions, and the resulting HRP-conjugated MAb 2-C10 was named HRP-2-C10.

### Development of B-ELISA.

ELISA plates (Nunc, Denmark) were coated with 100 μL of the purified rσB protein diluted in 0.05 M carbonated buffer (pH 9.6) overnight at 4°C. The plates were washed three times with PBS containing 0.05% Tween 20 (PBST) and then blocked with PBST containing 1% bovine serum albumin (BSA) (Sigma-Aldrich, St. Louis, MO, USA) at 37°C for 2 h. After washing, the plates could be used directly or kept at 4°C for 6 months. Antigen-coated 96-well plates were incubated with 50 μL of serum samples diluted 1:5 in diluent (PBST containing 1% BSA) at 37°C for 1 h. An equal volume (50 μL/well) of HRP-2-C10 in blocking buffer was added to all wells after the plates were washed three times with PBST. Then, the serum-HRP-conjugated MAb mixtures were incubated at 37°C for 1 h. After the plates were washed three times, 100 μL of the chromogenic substrate 3,3′,5, 5′-tetramethylbenzidine (TMB; 10 mg/mL) was added to each well. After the plates were incubated at 37°C for 10 min, color development was stopped by adding 50 μL of 2 M sulfuric acid per well. Each sample’s optical density (OD) value was measured using an ELISA microplate reader at 450 nm (Bio-Rad, Hercules, CA, USA). The results were converted to the PI by the following formula: PI (%) = [1 – (OD_450nm_ value of test serum sample/OD_450nm_ value of negative control serum)] × 100% (35). The percent inhibition values (PI) of 521 duck serum samples collected from different NDRV-free farms or nonimmune farms (serum I) were used to establish a negative cutoff value by adding three standard deviations to the mean PI.

The optimal coating concentration of the NDRV σB antigen (78, 156, 312.5, 625, 1,250, 2,500, and 5,000 ng/mL) and dilution of MAb 2-C10 (78, 156, 312.5, 625, 1,250, and 2,500 ng/mL) were determined by checkboard titration. The optimal serum dilution was determined by testing serial 2-fold dilutions of the serum (1:1, 1:2.5, 1:5, 1:10, 1:20, 1:40, 1:80, 1:160, and 1:320) against a fixed monoclonal antibody dilution (the optimal MAb dilution). The optimal dilution of each reagent was determined as the highest dilution that obtained the maximum PI difference between positive and negative serum (sera VI) (35).

### Validation of the B-ELISA.

A cross-blocking assay was performed by testing a known reference positive serum (serum III) against CDRV, DTMUV, DPV, GPV, MDPV, AIV (H9), DHAV-1, R. anatipestifer, and PA to evaluate the cross-reactivity of the newly developed B-ELISA.

Interassay and intra-assay reproducibility for the establishment of B-ELISA was assessed with eight NDRV serum samples (five positive samples and three negative samples) (serum VI) that had been verified by VNT. The CV was used to evaluate the intrarepeatability differences between different batches of precoated plates and the interrepeatability discrepancies of the same batch of precoated plates. Each sample was tested with three different plates on various occasions to determine the interassay CV, and three replicates within each plate were used to calculate the intra-assay CV.

### Comparisons of the B-ELISA and VNT.

A total of 212 serum samples (serum II) used in this study were tested for NDRV antibodies using the developed B-ELISA and the VNT, respectively. Results of both the B-ELISA and VNT were expressed as positive or negative for each sample to allow a qualitative comparison of the results.

### Statistical analysis.

Using the VNT as the gold standard, sensitivity and specificity analyses were calculated using the web-based MedCalc statistical software (https://www.medcalc.org/calc/diagnostictest.php). The agreement between the B-ELISA and VNT was calculated by the kappa values using SPSS software for windows, version 26.0 (IBM Corp., New York, USA). The figures were produced using Prism software 8.0.1 (GraphPad Software, Inc. LA Jolla, CA, USA). Differences between groups were considered statistically significant at *P < *0.05.
