# Supplementary material for: Development and Evaluation of a Monoclonal Antibody-Based Blocking Enzyme-Linked Immunosorbent Assay for the Detection of Antibodies against Novel Duck Reovirus in Waterfowl Species
Source: Microbiol Spectr. 2022 Nov 29;10(6):e02581-22. doi: 10.1128/spectrum.02581-22 (PMC9769907; doi:10.1128/spectrum.02581-22)
Supplement: Supplemental file 1 — Supplemental material. Download spectrum.02581-22-s0001.pdf, PDF file, 0.3 MB [file spectrum.02581-22-s0001.pdf]

Development and Evaluation of a Monoclonal Antibody-based Blocking ELISA for the Detection of Antibodies against Novel Duck Reovirus in Waterfowl Species

Tao Yun[a]*, Jionggang Hua[a], Weicheng Ye[a], Liu Chen[a], Zheng Ni[a], Yinchu Zhu[a], Cun Zhang[a]*

[a] State Key Laboratory for Managing Biotic and Chemical Threats to the Quality and Safety of Agro-products, Institute of Animal Husbandry and Veterinary Sciences, Zhejiang Academy of Agricultural Sciences, Hangzhou 310021, China

* Corresponding author:

Tao Yun

Email Address: yt-t@163.com

Cun Zhang

Email Address: zhangcun@aliyun.com

SUPPLEMENTARY MATERIALS

SUPPLEMENTARY TABLE

TABLE S1 Intra and Inter-assay repeatability test of 2-C10 based B-ELISA

Samples	Intra-assay			Inter-assay		
	Mean PI Value	SD	CV%	Mean PI Value	SD	CV%
1	93.43	1.28	1.37	92.62	1.50	1.62
2	85.15	1.68	1.97	86.29	2.11	2.44
3	85.52	2.62	3.06	86.78	1.79	2.06
4	65.03	2.49	3.82	66.42	2.41	3.63
5	58.41	0.77	1.32	56.32	2.25	4.00
6	8.05	0.57	7.02	7.75	0.46	5.94
7	3.51	0.23	6.69	3.56	0.31	8.69
8	13.78	0.58	4.22	14.04	0.68	4.87

SUPPLEMENTARY FIGURES

SUPPLEMENTARY FIGURE S1 Characterization of the rσB protein expressed in *E.coli* and the σB-specific mAb (2-C10) generated.

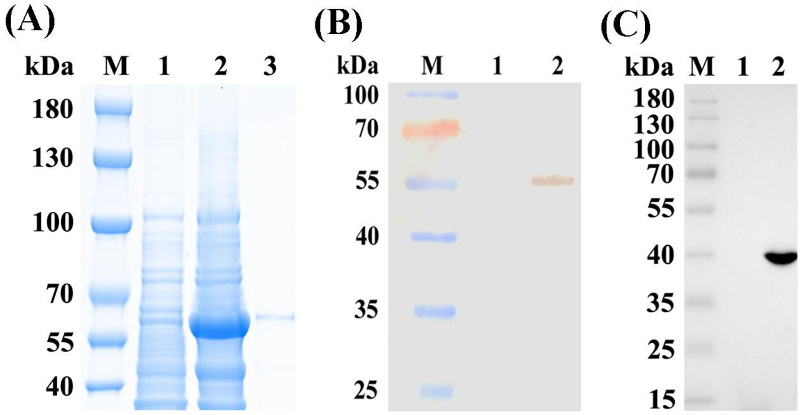

(A) Analysis of rσB protein by SDS-PAGE. Lane M, prestained protein molecular weight maker; Lane 1, crude extracts from *E. coli* before IPTG-induction;

Lane 2, crude extracts from *E. coli* after IPTG- induction; Lane 3, purified rσB protein.

(B) Western blot analysis of the purified recombinant SUMO-σB protein with duck anti-NDRV polyclonal serum. Lane M, prestained protein molecular weight maker; Lane 1, the purified His-SUMO-σB protein reacted with duck polyclonal serum; Lane 2, the whole bacterium lysates of BL21 (DE3) reacted with duck polyclonal serum.

(C) Western blot analysis of the σB of NDRV virion with 2-C10. Lane M, prestained protein molecular weight maker; Lane 1, Lysate supernatant of DF-1 cells uninfected with NDRV ZJ00M reacted with mAb 2-C10; Lane 2, Lysate supernatant of DF-1 cells infected with NDRV reacted with mAb 2-C10.

SUPPLEMENTARY FIGURE S2 Determination of the optimal dilution of coated protein, mAb and serum.

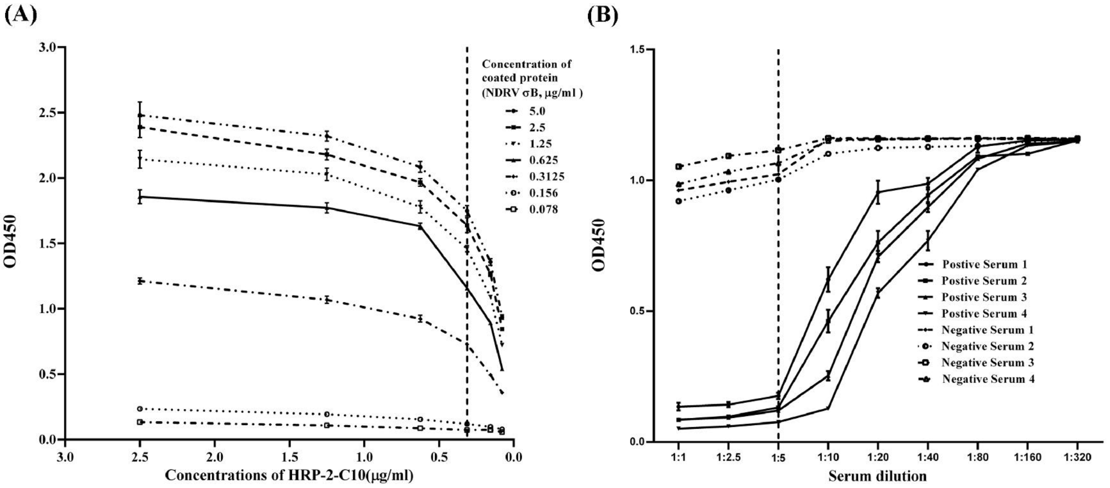

(A) Determination of optimal concentrations of coated protein (rσB) and horseradish peroxidase (HRP) conjugation of mAb 2-C10 (HRP-2-C10). The optimal concentration of the coated protein (0.3125 μg/ml) and HRP-2-C10 (0.625 μg/ml) was chosen, the OD450 value of B-ELISA was always around 1.2, which was on the titration curve (linear range) to obtain optimal inhibition.

(B) Determination of an optimal dilution of serum. Serial two-fold dilutions of

negative and positive control sera were reacted with the optimal mAb concentration (1:5) in B-ELISA. To minimize the volume of serum required, a serum dilution of 1:5 was chosen as the preferred dilution that performed only slightly better. Data were presented as means ± SD from at least three independent experiments.

SUPPLEMENTARY FIGURE S3. Cross-reactivity of B-ELISA with other viruses and bacteria.

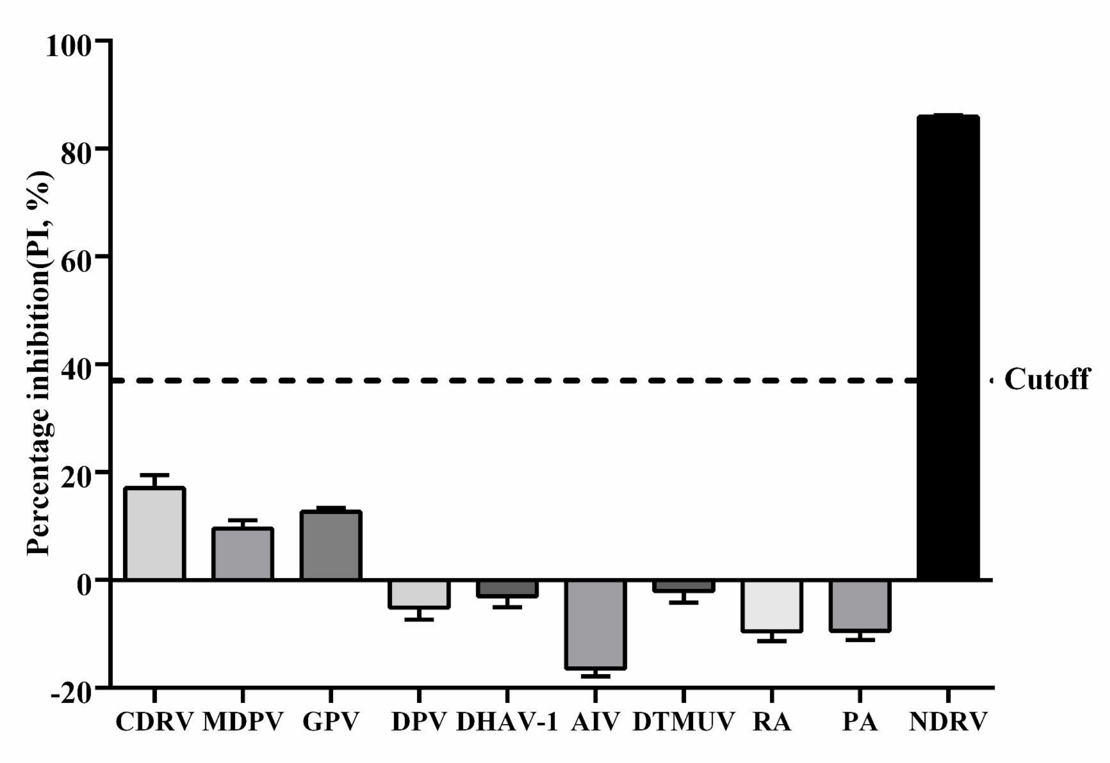

AIV: avian influenza virus subtypes H5 and H9, CDRV: classical DRV, DHAV-1: duck hepatitis A virus, DPV: duck plague virus, DTMUV: duck tembusu Virus, GPV: goose parvovirus, MDPV: Muscovy duck parvovirus, PA: *Pasteurella anatipestifer*, RA: *Riemerella anatipestifer*. The dashed line represents the cut-off value (37.01). Data were presented as means ± SD from at least three independent experiments.
